# Surface Termination Conversion during SrTiO_3_ Thin Film Growth Revealed by X-ray Photoelectron Spectroscopy

**DOI:** 10.1038/srep11829

**Published:** 2015-07-20

**Authors:** Christoph Baeumer, Chencheng Xu, Felix Gunkel, Nicolas Raab, Ronja Anika Heinen, Annemarie Koehl, Regina Dittmann

**Affiliations:** 1 Peter Gruenberg Institute and JARA-FIT, FZ Juelich, D-52425 Juelich, Germany; 2 Institut fuer Werkstoffe der Elektrotechnik II, RWTH Aachen University, D-52074 Aachen, Germany

## Abstract

Emerging electrical and magnetic properties of oxide interfaces are often dominated by the termination and stoichiometry of substrates and thin films, which depend critically on the growth conditions. Currently, these quantities have to be measured separately with different sophisticated techniques. This report will demonstrate that the analysis of angle dependent X-ray photoelectron intensity ratios provides a unique tool to determine both termination and stoichiometry simultaneously in a straightforward experiment. Fitting the experimental angle dependence with a simple analytical model directly yields both values. The model is calibrated through the determination of the termination of SrTiO_3_ single crystals after systematic pulsed laser deposition of sub-monolayer thin films of SrO. We then use the model to demonstrate that during homoepitaxial SrTiO_3_ growth, excess Sr cations are consumed in a self-organized surface termination conversion before cation defects are incorporated into the film. We show that this termination conversion results in insulating properties of interfaces between polar perovskites and SrTiO_3_ thin films. These insights about oxide thin film growth can be utilized for interface engineering of oxide heterostructures. In particular, they suggest a recipe for obtaining two-dimensional electron gases at thin film interfaces: SrTiO_3_ should be deposited slightly Ti-rich to conserve the TiO_2_-termination.

The extraordinary electrical and magnetic properties emerging at oxide interfaces provide an intriguing platform for creating new functionalities^1^,^2^. For most of the fascinating properties observed for thin films or heterointerfaces, the termination of the films and the substrates plays a dominant role. This has been observed for the electronic properties of high-mobility two-dimensional electron gases at the interface of insulators such as LaAlO_3_/SrTiO_3_^3^,^4^,^5^,^6^,^7^, LaVO_3_/SrTiO_3_^8^, and NdGaO_3_/SrTiO_3_^9^,^10^ and high-T_*c*_ superconductivity in cuprate interfaces^11^. The same is true for magnetic oxides such as LaMnO_3_/SrTiO_3_ superlattices and SrRuO_3_ and La_2/3_Sr_1/3_MnO_3_ thin films, which show desirable magnetic properties and half-metallicity, which can form the basis for magnetic tunnel junctions and spintronics^12^,^13^,^14^,^15^,^16^,^17^. Moreover, these properties intrinsically depend on the thin film stoichiometry^18^,^19^,^20^,^21^. Interestingly, the interface of LaAlO_3_ with SrTiO_3_ thin films has been observed to be increasingly insulating for pulsed laser deposited, homoepitaxial SrTiO_3_ thin films of increasing thickness, which appears to be connected to defect formation during growth^6^,^7^,^21^.

Since recent reports even observed unintentional change of surface termination in the molecular beam epitaxy of layered Sr_*n*+1_Ti_*n*_O_*n*+1_ Ruddlesden-Popper phases^22^, one could reasonably assume that the loss of conductivity in these interfaces might also be caused by a growth induced surface termination conversion. It is therefore imperative to precisely determine and control both stoichiometry and surface termination of these films and their substrates to tailor their properties and their interplay.

To date, both quantities are typically determined separately using established non-destructive techniques such as X-ray photoelectron diffraction^23^,^24^, ion scattering spectroscopy^25^, coaxial impact collision ion scattering spectroscopy^26^, scanning transmission electron microscopy^4^,^27^, and scanning probe microscopy^5^,^28^,^29^,^30^ for the termination and Rutherford backscattering spectrometry as well as X-ray photoelectron spectroscopy (XPS) for the stoichiometry^18^,^19^,^20^. While XPS presents the most accessible tool for the determination of thin film stoichiometry, the impact of the termination layer is usually not taken into account when the films are compared to reference data, although Zhang *et al*. pointed out that different terminations of single crystals lead to differences in the intensity ratios by as much as 17%, which can lead to dramatic errors in the stoichiometry estimate^31^.

At the same time, as this report will demonstrate, analysis of the angle dependence inherent to this termination-dependent X-ray photoelectron intensity renormalization provides a unique tool to directly determine the substrate or thin film termination and stoichiometry simultaneously. This can be achieved in a fast measurement and simple data analysis without the need for high angular resolution or complex modeling. We acquired a simple analytical model which accurately describes the angle dependent cation X-ray photoelectron intensity ratio of SrTiO_3_ single crystals with different terminations. In a second step, we use this model to demonstrate that during homoepitaxial SrTiO_3_ growth, excess cations are indeed consumed in a self-organized surface termination conversion before cation defects are incorporated into the film. This understanding of growth-induced surface termination conversion during pulsed laser deposition explains the suppression of the electron gas detected at LaAlO_3_/SrTiO_3_ thin film interfaces fabricated subsequently. These insights can be utilized for precise interface engineering, providing a new tool to tailor the interfacial properties.

## Results

### Analytical consideration

**Figure 1:**
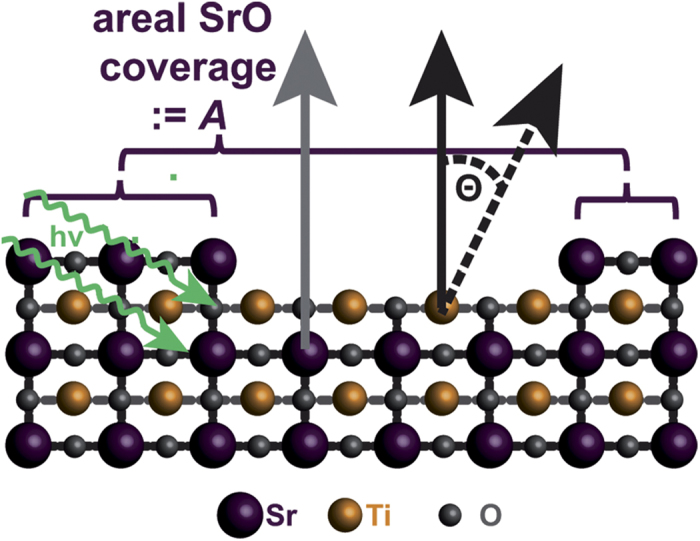
Photoemission process in SrTiO_3_. Schematic illustration of the photoemission process from SrTiO_3_ single crystals. Black and grey arrows represent electrons emitted at Ti and Sr ions, respectively.

In order to quantitatively describe the cation photoelectron intensities, we first consider a SrTiO_3_ single crystal composed of equally spaced layers of TiO_2_ and SrO under X-ray illumination as shown in Fig. 1. For regions of perfect TiO_2_-termination, Ti^2p^ photoelectrons excited in the first monolayer can leave the crystal unattenuated with intensity 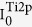, while Ti^2p^ photoelectrons from deeper layers are attenuated by each SrTiO_3_ unit cell they have to penetrate. The intensity contribution 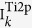 from each layer *k* (*k* = 0,1,2, …) can then be expressed as


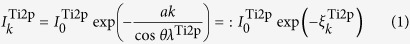


with the lattice parameter *a* = 3.905 Å, the inelastic mean free path (IMFP) *λ*^Ti2p^ for photoelectrons at the characteristic kinetic energy and the photoemission angle *θ*. Sr^3d^ electrons, however, are even attenuated if they are excited within the first unit cell, since they have to penetrate the terminating TiO_2_-monolayer. Consequently, Sr^3d^ photoelectrons from layer *k* are attenuated by *k* + 0.5 unit cells and their intensity can be expressed as


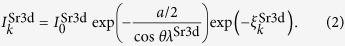


Following the same considerations for regions of perfect SrO-termination, we arrive at the following expressions for the total intensities:


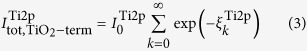



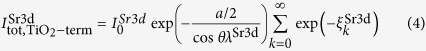



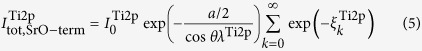



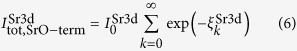


Equations (3) and (4) represent the total intensity of Ti^2p^ and Sr^3d^ photoelectrons for perfect TiO_2_-termination, respectively, while equations (5) and (6) represent the total intensity of Ti^2p^ and Sr^3d^ photoelectrons for perfect SrO-termination. If we now consider a mixed termination, which can be imagined as a perfectly TiO_2_-terminated SrTiO_3_ single crystal with an areal coverage *A* of a SrO monolayer, as depicted in Fig. 1, we arrive at


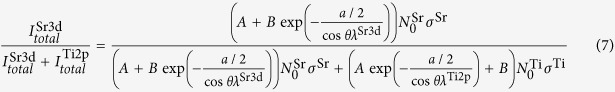


with the total atomic concentrations 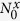 and the sensitivity factor *σ*^*x*^ for cation *x* and the areal coverage of TiO_2_-termination *B* = 1 − *A*. The transmission functions as well as the energy dependence of 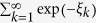 are included in the calibration of the sensitivity factors. This expression now describes the cation photoelectron intensity ratio as a function of only the photoemission angle *θ* for a given surface termination and stoichiometry. While this model does not include the positions of scattering atoms within the crystal, which are the essential part of the complex photoelectron diffraction models, it treats the crystal semi-continuously. This leads to much easier fitting of the model to experimental data and does not require a high angular resolution, as will be shown in the following paragraphs. We note that the stoichiometry of the crystal is represented in equation (7) through the total atomic concentrations 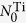 and 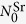. This expression is valid as long as the approximate position of each species (i.e. the depth *k*, at which the photoelectron is excited) remains the same as in the stoichiometric crystal. It is therefore evident that our model accurately represents crystals with homogeneously distributed point defects, while quantitative errors may occur in the descriptions of highly non-stoichiometric SrTiO_3_ crystals, which have been shown to possess extended defects like Ruddlesden-Popper phases^32^. In two worst case scenarios, if a SrO double layer developed close to the surface of the crystal, or if the entire crystal possessed a Ruddlesden-Popper-type layered structure, we end up with a relative error of below 3.5% as shown in Supplementary Note 1, Supplementary Fig. 1 and Supplementary Table 1.

### Termination determination in SrTiO_3_ single crystals

**Figure 2:**
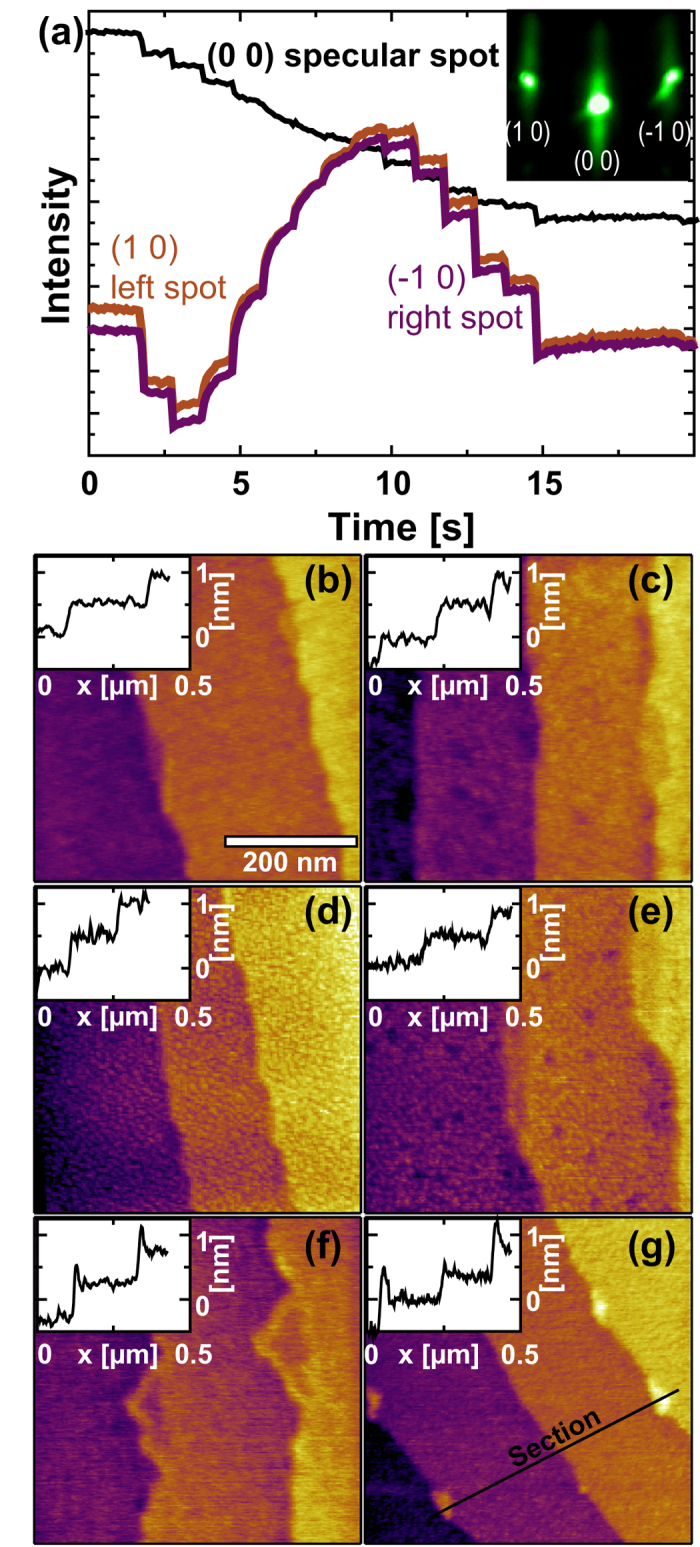
Systematic surface termination variation in SrTiO_3_ single crystals. (**a**) Representative RHEED intensity during SrO deposition. Inset: Exemplary RHEED pattern after SrO growth (14 pulses). (**b**–**g**) AFM morphology after deposition of 2, 4, 6, 8, 10, and 12 pulses of SrO, respectively. Step terraces are of unit cell height (≈4 Å). Insets: Representative line profiles from the AFM scans along sections like the one depicted in (**g**).

Sub-monolayer films of SrO and films exceeding one monolayer to a small extent were deposited *via* PLD on substrates obtained from one TiO_2_-terminated SrTiO_3_ single crystal (see methods for details). The film growth was monitored using reflection high energy electron diffraction (RHEED) along <110> direction, as shown for an exemplary deposition of 14 pulses of SrO in Fig. 2(a). While the intensity of the specular spot continuously decreases with the amount of SrO deposited on the surface, the intensities of the (10) and (−10) 1^st^ order diffracted electrons have a maximum once the coverage of approximately one complete layer of SrO on the SrTiO_3_ crystal is reached after 9 pulses. This relative change of RHEED intensity between the specular point (00) and the (10) and (−10) points has also been observed in MBE-growth of SrO monolayers. For more than one monolayer of SrO one expects the additional appearance of half-integer RHEED streaks^22^. Therefore, the absence of such streaks is an additional indication that none of the SrO films with up to 9 pulses exceeded the thickness of 1 monolayer.

The morphology evolution after the deposition of increasing amounts of SrO was monitored with an ultra-high vacuum AFM after *in-situ* ultra-high vacuum transfer from the PLD system. Each image was taken at room temperature after quenching the sample directly after the PLD process. The surface morphology for different amounts of SrO termination is depicted in Fig. 2(b–g). The deposition of less than one monolayer of SrO results in the creation of small islands. The SrTiO_3_ step terraces remain sharp and no islands of one SrTiO_3_ unit cell height or more can be observed, confirming that only the surface termination of the crystal changes. Only when ten or more pulses of SrO are deposited, higher islands begin to form at the step edges [compare the line profiles in the insets of Fig. 2(f,g)]; we assume that SrO starts to nucleate as a separate phase. These AFM scans support the conclusion from the RHEED analysis that the deposition of 9 pulses of SrO results in the deposition of a nearly complete SrO terminating layer on previously perfectly TiO_2_-terminated SrTiO_3_.

**Figure 3:**
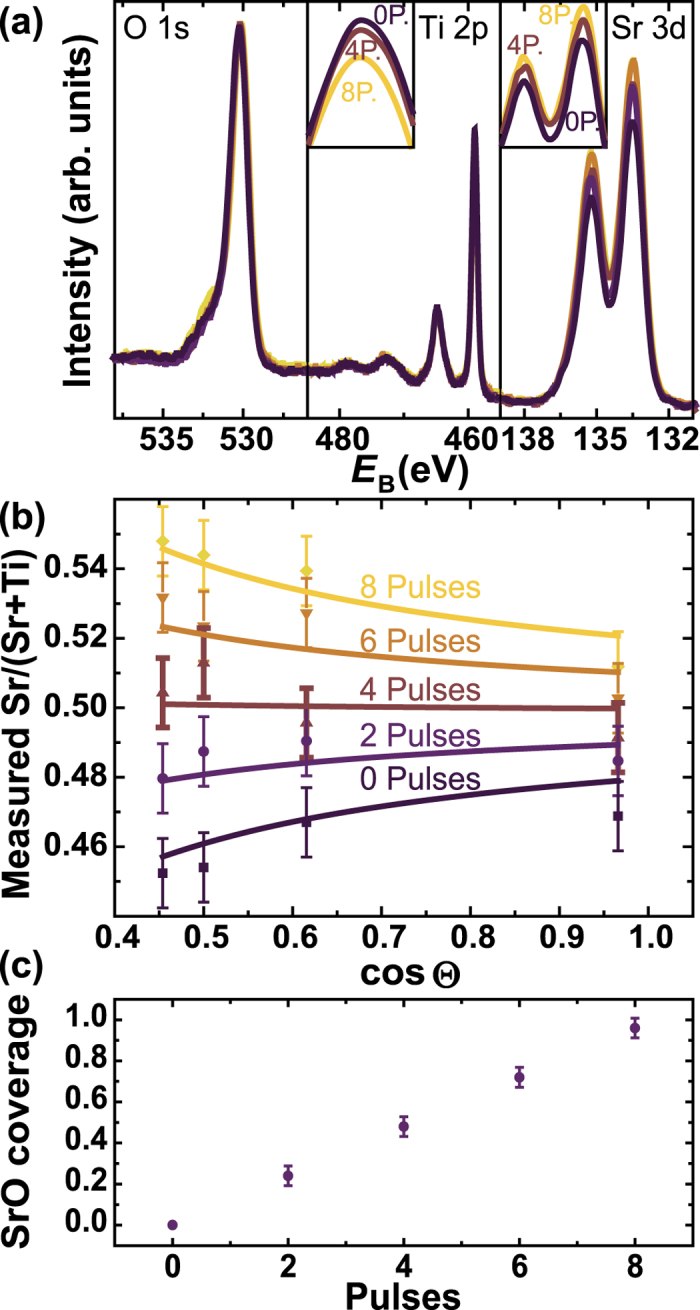
XPS analysis and termination determination of SrTiO_3_ single crystals. (**a**) Representative XPS spectra for SrTiO_3_ crystals with increasing amounts of SrO coverage normalized to the O^1s^ peak intensity. The Ti^2p^ and Sr^3d^ peaks have the same shape and binding energy for each sample, indicating the absence of a second phase. Insets: Zoom-in on the peaks for films with 0, 4 and 8 pulses of SrO. (**b**) Measured cation ratios (data points) as a function of the photoemission angle and fits (solid lines) according to equation (7). (**c**) SrO coverage extracted from the fits in (**b**).

After AFM analysis, all samples with up to one monolayer of SrO (i.e. samples with 0, 2, 4, 6, and 8 pulses) were characterized by XPS at various photoemission angles. Again, the samples were transferred from one analysis tool to the next in ultra-high vacuum. For each angle, the O^1s^, Ti^2p^ and Sr^3d^ peaks were measured, indicating relative intensity changes between Ti^2p^ and Sr^3d^ but no second phases, as is apparent from the identical peak shapes [Fig. 3(a)]. The apparent atomic concentration was extracted for each angle based on the intensity ratios as shown in Fig. 3(b). As expected, TiO_2_-terminated samples show a measured Sr/(Sr + Ti) ratio of less than 0.5 with a further decrease towards more surface sensitive measurements. Mixed termination leads to a rather constant cation ratio, while SrO-termination leads to the opposite trend observed for the TiO_2_-termination.

For quantitative analysis, this angle dependence was fitted according to equation (7). The cation ratio of each sample was constrained to ideal stoichiometry (i.e. 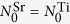) and the sensitivity factors were calibrated during the fitting routine. The IMFPs were constrained to literature values of *λ*^Ti2p^ = 21 Å and *λ*^Sr3d^ = 26 Å^33^ and the surface termination *A* - the only physical variation between the samples - was allowed to vary under the constraint of linear increase of SrO-termination with the number of pulses. The measured, angle dependent cation ratios and the fits are shown in Fig. 3(b), indicating an excellent agreement of our analytical model with the measurement. The generality of our model is underlined by the fact that opening the constraint on IMFP and the linear increase in SrO coverage resulted in very similar IMFP and *A* values. The results of the fits are summarized in Fig. 3(c), where a linear increase of SrO-termination from 0% to nearly complete coverage is apparent, which agrees very well with the RHEED and AFM results. We can therefore conclude that the model described in equation (7) can accurately describe the termination-induced angle dependence of the photoelectron intensity ratios and that such a straightforward measurement (total measurement time of only 3 hours for each sample) can determine the fraction of each terminating layer with high accuracy. It is furthermore obvious that although all of these samples have an ideal cation stoichiometry of 50% Sr and 50% Ti, the differences in surface termination would lead to a measurement error of several percent points even for close-to-normal photoemission angles if the termination effects were not included in the analysis of XPS data.

### Termination conversion during SrTiO_3_ thin film deposition

Since the determination of the termination layer was performed here with a well-established tool for stoichiometry determination, thin film stoichiometry can be determined simultaneously with the termination. In fact, the potential termination-induced error in the measured stoichiometry can be avoided through analysis of the angle dependent cation intensity ratio. To demonstrate this procedure, we prepared four homoepitaxial SrTiO_3_ thin films with varying laser fluence, which presents a well-known tool for the fabrication of thin films with different cation ratios^18^,^19^,^20^ and analyzed them *via* AFM and XPS after ultra-high vacuum transfer. 20 nm films were obtained in a similar PLD process as described above on multiple pieces of one TiO_2_-terminated SrTiO_3_ single crystal with the same miscut as used before.

**Figure 4:**
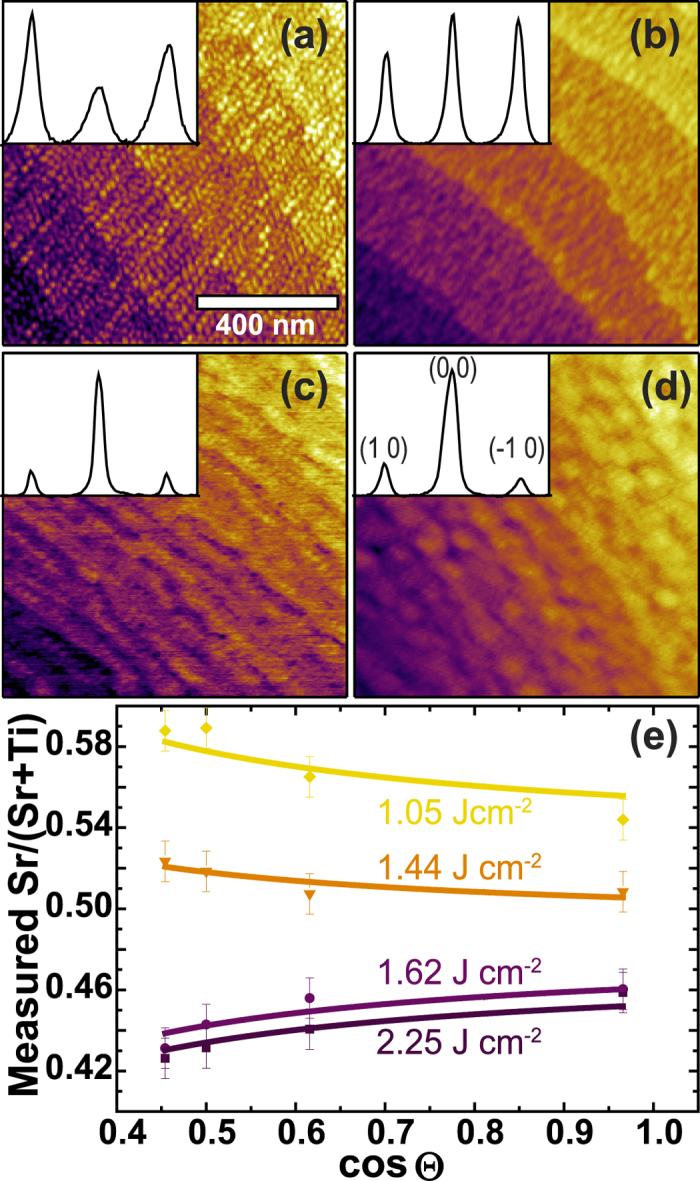
Termination and stoichiometry determination for SrTiO_3_ thin films. (**a**–**d**) AFM morphology of 20 nm homoepitaxial SrTiO_3_ thin films grown with a laser fluence of 1.05, 1.44, 1.62 and 2.25 J·cm^−2^, respectively. Step terraces are of unit cell height (≈4 Å). Inset: Line profiles extracted from the RHEED pattern after growth. For each case, only the specular spot and 1^st^ order diffracted electrons are detected (each peak is labeled exemplary in (**d**)). (**e**) Measured cation ratios (data points) as a function of the photoemission angle and fits (solid lines) according to equation (7).

The surface morphology for each film is shown in Fig. 4(a–d). For the films with lower fluences, the vicinal step surface structure of the substrate is well preserved. For films with higher fluences, the step edges are not as apparent, but the surfaces are equally smooth with a root-mean-square roughness below 0.25 nm. RHEED intensity profiles were obtained through line-by-line integration of the RHEED intensity perpendicular to the diffraction streaks of (−10), (00) and (10). These profiles qualitatively indicate an increase of SrO-termination fraction with decreasing laser fluence [insets of Fig. 4(a–d)], which is apparent from the increasing intensity of the 1^st^ order diffracted electrons compared to the specular spot [compare RHEED intensity plot in Fig. 2(a) and Ref. 22].

This qualitative observation is further supported by angle dependent XPS measurements [Fig. 4(e)], which show cation ratio trends indicative of SrO-termination (increase of the measured Sr/(Sr + Ti) ratio for increasingly surface sensitive measurements) for films deposited with low laser fluences and the opposite trend for higher fluences. Quantitative values for the termination and stoichiometry can be extracted by fitting the angle dependent XPS-measurements according to equation (7). For these fits, which are also shown in Fig. 4(e), the IMFPs and sensitivity factors were fixed to the values determined for single crystals and the amount of SrO-termination and the atomic concentration of Sr, 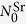, were allowed to vary. The IMFP values are typically calculated for stoichiometric samples; given the weak dependence of the electron inelastic mean free paths on the stoichiometry or possible defects, however, utilizing these literature values for slightly non-stoichiometric samples is justified. The fit parameters for the thin film stoichiometry and termination are shown in Table 1.

From the values for the SrTiO_3_ thin film stoichiometry and termination extracted simultaneously through these fits, it is obvious that despite appreciable error bars the previously reported qualitative trends of Ti-rich films for high fluences, close to stoichiometric films for medium fluences and Sr-rich films for low fluences holds^18^,^20^. We would like to emphasize, however, that the cation ratio difference of films with a similar stoichiometry but different termination layers can be heavily underestimated or overestimated if the termination effect is not considered. This is obvious for our samples grown at 1.44 J·cm^−2^ and 1.62 J·cm^−2^. They exhibit a cation ratio difference of just roughly one percent point, but they could be misinterpreted to possess a difference of five percent points even for normal emission.

Even more importantly, our results add the additional information of preferential SrO-termination for films grown at lower fluences, for Sr-rich but also for stoichiometric films. Since the substrates were perfectly TiO_2_-terminated, this means that during the growth process, excess Sr ions are consumed in a self-organized surface termination conversion from TiO_2_-termination to SrO-termination on a nearly stoichiometric film rather than through incorporation of substantial amounts of defects into the film. This is most apparent for the sample grown at 1.44 J·cm^−2^, which is stoichiometric but possesses predominant SrO-termination. Only upon further decrease of the laser fluence (1.05 J·cm^−2^) we observe significant incorporation of this Sr excess into the film. We therefore infer that this incorporation of large amounts of defects starts after the surface termination conversion is completed or if rather large amounts of excess Sr are present.

**Figure 5:**
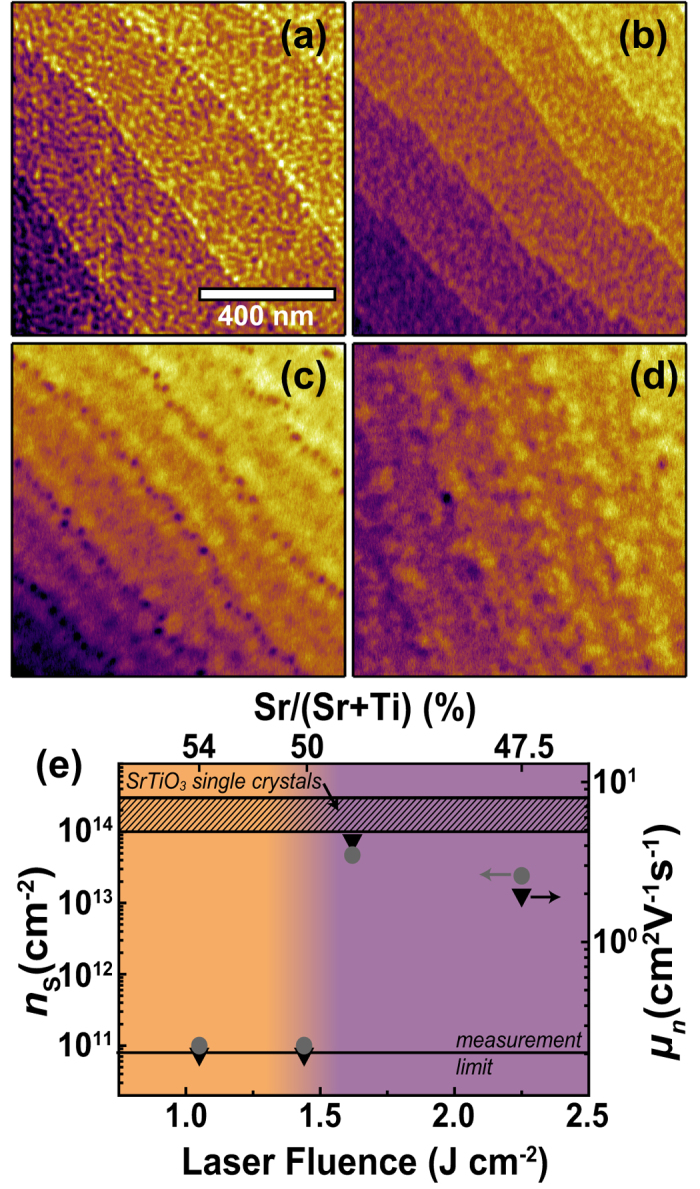
Characterization of LaAlO_3_/SrTiO_3_ thin film heterointerfaces. (**a**–**d**) AFM morphology of 10 unit cells heteroepitaxial LaAlO_3_ thin films grown on the films shown in Fig. 4. Step terraces are of unit cell height (≈4 Å). (**e**) Carrier density (grey dots) and electron mobility values (black triangles) extracted from Hall measurements as a function of laser fluence. The approximate SrTiO_3_ stoichiometry is indicated through the top axis. The surface termination is indicated with a color gradient from complete SrO-termination (orange) to complete TiO_2_-termination (violet). The carrier density and mobility range expected for LaAlO_3_/SrTiO_3_ interfaces fabricated with SrTiO_3_ single crystals is indicated with the shaded area. For the SrO-terminated SrTiO_3_ films, the sheet resistance of the resulting heterostructures is above the measurement limit, which inhibits the extraction of quantitative values for the carrier density and mobility.

To demonstrate the strong impact this surface termination conversion has upon the properties of oxide heterostructures, we fabricated LaAlO_3_/SrTiO_3_ interfaces on each of these SrTiO_3_ films shown in Fig. 4 through the deposition of 10 unit cells of LaAlO_3_ *via* PLD. The surface morphology for each film is shown in Fig. 5(a–d). For each film, layer-by-layer growth mode was observed and the surface structure of the underlying SrTiO_3_ film is well preserved. For the films grown on (partially) SrO-terminated SrTiO_3_, the vicinal step surface structure of the substrate is still apparent, while the films grown on TiO_2_-terminated SrTiO_3_ do not show these step edges. However, these films are still fairly smooth with a root-mean-square roughness below 0.34 nm.

In line with our findings on the termination conversion during SrTiO_3_ growth, the electrical properties for the resulting LaAlO_3_/SrTiO_3_ interfaces differ substantially, which is evident from the results of room temperature Hall measurements shown in Fig. 5(e). Both heterostructures fabricated on (partially) SrO-terminated SrTiO_3_ films possess a sheet resistance above the measurement limit of approximately 10 MΩ, corresponding to a suppression of the electron gas at the interface. In contrast, both heterostructures fabricated on TiO_2_-terminated SrTiO_3_ films exhibit an electron gas with properties close to the typical values for LaAlO_3_/SrTiO_3_ interfaces fabricated on TiO_2_-terminated SrTiO_3_ single crystals. The heterostructure fabricated on the SrTiO_3_ film with the most significant Ti excess (grown at 2.25 J·cm^−2^) possesses a slight increase of the sheet resistance due to a reduction of the electron density and mobility. The reduced electron mobility is indicative for incorporated defects^21^ and the reduced carrier density hints at electron trapping at Sr vacancies, which are likely present in Ti-rich films^34^.

## Discussion

The observation of surface termination conversion, which appears to be one major mechanism for excess cation consumption during thin film growth, can explain the reduced carrier density and even the suppression of the electron gas at LaAlO_3_/SrTiO_3_ thin film interface observed for the heterostructures fabricated in this report and in literature^6^,^7^. It is well known that the AlO_2_/SrO/TiO_2_ interface is insulating due to compensation of the interfacial dipole by an atomic interface reconstruction^4^. Indeed, for both films grown on preferential SrO-termination, we observe the complete suppression of the electron gas, even for a stoichiometric SrTiO_3_ film, while the heterostructures shown on TiO_2_-terminated SrTiO_3_ films remain very conductive. Judging from the surface morphology, one would not have anticipated the stoichiometric SrTiO_3_ film with perfect step terraces to result in an insulating interface while rougher films grown with Ti excess exhibit excellent conductivity. Only the transition from TiO_2_-termination to SrO-termination during the film growth observed and quantified here through the angle dependent XPS measurements can account for this behavior and must therefore carefully be avoided in the fabrication of SrTiO_3_ thin films for conducting interfaces. At the same time, it is equally important to control the SrTiO_3_ thin film stoichiometry, as growth-induced defects in non-stoichiometric SrTiO_3_ lead to a reduction of the electron mobility as observed previously and confirmed here^21^. Both termination control and stoichiometry control can be achieved through the careful selection of the proper deposition parameters for the chosen film thickness. For the specific case of SrTiO_3_ thin films for conductive interfaces, slightly Ti-rich deposition parameters should be selected to conserve the TiO_2_-termination. Significant Ti-excess, however, must be inhibited to avoid electron scattering through defects as much as possible.

In conclusion, we have introduced and utilized an analytical model for the angle-dependent cation photoelectron intensity ratio taking into account both the termination layer and the stoichiometry of the thin film or single crystal. Fitting angle-dependent XPS data with this model allows the extraction of both quantities simultaneously. As an example, we quantified the resulting termination after sub-monolayer pulsed laser deposition of SrO on TiO_2_-terminated SrTiO_3_ single crystals and determined both stoichiometry and termination for homoepitaxial SrTiO_3_ thin films grown with different laser fluences. For the case of thin films, we observe a self-organized surface termination conversion from TiO_2_-termination to SrO-termination for films grown at lower laser fluences. This termination conversion can be responsible for insulating properties of interfaces between polar perovskites and SrTiO_3_ thin films and must therefore be avoided by the proper selection of growth parameters and careful characterization of the thin films. Our analytical model therefore presents a novel and straightforward tool for the process control of oxide heterointerface fabrication and sheds light onto the properties of LaAlO_3_/SrTiO_3_ thin film interfaces.

## Methods

SrO was deposited *via* PLD on substrates obtained from one SrTiO_3_ single crystal (Crystec GmbH, Germany), which was HF-etched for complete TiO_2_-termination^28^. A polycrystalline SrO_2_ target was ablated with a KrF excimer laser (*λ* = 248 nm) with a repetition rate of 1 Hz and a laser fluence of 1.3 J·cm^−2^ (spot size 2 mm^2^) at a target-substrate distance of 44 mm in an atmosphere of 10^−7^ mbar O_2_; the substrate temperature was 800 ºC. The film growth was monitored using reflection high energy electron diffraction (RHEED, akSA400 system with an integration time of 8 ms).

For the deposition of SrTiO_3_ thin films, a single crystalline SrTiO_3_ target was ablated with a repetition rate of 1 Hz and a laser fluence of 1.05–2.25 J·cm^−2^ in an atmosphere of 0.1 mbar O_2_; the substrate temperature was 800 ° C.

For the deposition of LaAlO_3_ thin films, a single crystalline LaAlO_3_ target was ablated with a repetition rate of 1 Hz and a laser fluence of 1.9 J·cm^−2^ in an atmosphere of 10^−4^ mbar O_2_; the substrate temperature was 700 ° C.

UHV AFM was performed on the SrTiO_3_ thin films and single crystals with different termination using an Omicron VT SPM operated in contact mode with a single crystalline diamond tip with a nominal tip radius of 10 nm (Advanced Diamond Technologies). The morphology of LaAlO_3_ thin films was detected *ex-situ* using a Surface Analysis Systems Picostation AFM in tapping mode.

The XPS measurements were performed with a PHI 5000 Versa Probe (Physical Electronics Inc., USA) with Al K_*α*_ X-ray illumination, a pass energy of 29.35 eV and at various photoemission angles using electron neutralization.

## Additional Information

**How to cite this article**: Baeumer, C. *et al.* Surface Termination Conversion during SrTiO_3_ Thin Film Growth Revealed by X-ray Photoelectron Spectroscopy. *Sci. Rep.* **5**, 11829; doi: 10.1038/srep11829 (2015).

## Supplementary Material

Supplementary Information

## Figures and Tables

**Table 1:** Thin film surface termination and stoichiometry.

**Fluence (J.cm^−2^)**	**SrO-term, *A***	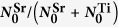
1.05	100%	54.0 ± 2.0%
1.44	76%	49.8 ± 1.6%
1.62	0%	48.5 ± 1.6%
2.25	0%	47.7 ± 1.6%
