# Supplementary material for: Surface Termination Conversion during SrTiO3 Thin Film Growth Revealed by X-ray Photoelectron Spectroscopy
Source: Sci Rep. 2015 Jul 20;5:11829. doi: 10.1038/srep11829 (PMC4507138; doi:10.1038/srep11829)
Supplement: Supplementary Information [file srep11829-s1.pdf]

Surface Termination Conversion during $SrTiO_3$ Thin Film Growth Revealed by X-ray Photoelectron Spectroscopy

Christoph Baeumer[1,*], Chencheng Xu[1], Felix Gunkel[1], Nicolas Raab[1], Ronja Anika Heinen[1], Annemarie Koehl[1], and Regina Dittmann[1]

[1]Peter Gruenberg Institute and JARA-FIT, FZ Juelich, D-52425 Juelich, Germany

*c.baeumer@fz-juelich.de

Supplementary Notes

Supplementary Note 1

To demonstrate the effect extended defects may have on the determination of the cation ratio using equation 7 from the main text, we analyzed 2 extreme cases of extended defects. For this purpose, we analytically calculated the measured cation intensity ratios of the crystals containing the extended defect and fitted the resulting data points with our model for the perfect crystal. As shown in Supplementary Fig. 1a, we started with a stoichiometric $SrTiO_3$ crystal with SrO termination, which can be described accurately using equation 7 of the main text. For the first case of extended defects which might be induced by non-stoichiometric growth, we inserted one additional layer of SrO below the first unit cell (Supplementary Fig. 1b). This could correspond to a 5 nm $SrTiO_3$ film with 2 % Sr-excess, if the entire Sr-excess were consumed in the formation of this Ruddelsden-Popper-type defect. Alternatively, this can be regarded as a Ruddelsden-Popper-phase film with the stoichiometry $Sr_{14}Ti_{13}O_{40}$. We chose to incorporate this defect as close to the surface as possible to demonstrate the most severe effect such extended defects my have on the measured film stoichiometry. Note that a SrO double layer at the surface of the $SrTiO_3$ crystal is an unrealistic scenario, as it is energetically unfavorable.[1] In a second step, we inserted further SrO layers into the crystal, yielding a Ruddelsden-Popper-phase film with the stoichiometry Sr_2TiO_4 (Supplementary Fig. 1c). This film can hardly be regarded as a non-stoichiometric $SrTiO_3$ film, but we include it in our analysis to demonstrate the maximum effect extended defects like Ruddelsden-Popper-phases can have. For both cases described above, we calculated the relative cation photoemission intensities layer by layer, in a similar way as described in the main text. For the $Sr_{14}Ti_{13}O_{40}$ film we obtain

$$I_0^{\mathrm{Sr3d}} = I_0^{\mathrm{Sr3d}} \tag{1}$$

$$I_1^{\mathrm{Ti2p}} = I_0^{\mathrm{Ti2p}} \exp\left(-\frac{a}{2\cos\theta\lambda^{\mathrm{Ti2p}}}\right) \tag{2}$$

$$I_2^{\mathrm{Sr3d}} = I_0^{\mathrm{Sr3d}} \exp\left(-\frac{a}{\cos\theta\lambda^{\mathrm{Sr3d}}}\right) \tag{3}$$

$$I_3^{\mathrm{Sr3d}} = I_0^{\mathrm{Sr3d}} \exp\left(-\frac{3a}{2\cos\theta\lambda^{\mathrm{Sr3d}}}\right) \tag{4}$$

$$I_4^{\mathrm{Ti2p}} = I_0^{\mathrm{Ti2p}} \exp\left(-\frac{2a}{\cos\theta\lambda^{\mathrm{Ti2p}}}\right) \tag{5}$$

$$I_5^{\mathrm{Sr3d}} = I_0^{\mathrm{Sr3d}} \exp\left(-\frac{5a}{2\cos\theta\lambda^{\mathrm{Sr3d}}}\right) \tag{6}$$

$$\ldots \tag{7}$$

for the intensity I_k^{Sr3d} or I_k^{Ti2p} from layer k. Summing over all layers of a 5 nm film, we arrive at the angle-dependent intensities indicated in Supplementary Fig. 1d.

For Sr_2TiO_4 we obtain

$$I_k^{\mathrm{Sr3d,layer\ 1}} = I_0^{\mathrm{Sr3d}}(-\xi_k'^{\mathrm{Sr3d}}) \tag{8}$$

$$I_k^{\mathrm{Sr3d,layer\ 2}} = I_0^{\mathrm{Sr3d}} \exp\left(-\frac{a'/3}{\cos\theta\lambda^{\mathrm{Sr3d}}}\right)\exp(-\xi_k'^{\mathrm{Sr3d}}) \tag{9}$$

$$I_k^{\mathrm{Ti2p,layer\ 3}} = I_0^{\mathrm{Ti2p}} \exp\left(-\frac{2a'/3}{\cos\theta\lambda^{\mathrm{Ti2p}}}\right)\exp(-\xi_k'^{\mathrm{Ti2p}}) \tag{10}$$

for the intensity $I_k^{\mathrm{Sr3d,layer\ 1}}$, $I_k^{\mathrm{Sr3d,layer\ 2}}$ or $I_k^{\mathrm{Ti2p,layer\ 3}}$ from the first and second SrO layer and the TiO_2 layer from unit cell k, respectively, if we define the Sr_2TiO_4 out-of-plane lattice parameter $a' \approx 6$Å and $\xi_k'^{\mathrm{Ti2p}} = \frac{a'k}{\cos\theta\lambda^{\mathrm{Ti2p}}}$. Summing over all layers of a 5 nm film, we reach at the angle-dependent intensities indicated in Supplementary Fig. 1d.

We now treat these analytically determined cation intensity ratios for the defective $SrTiO_3$ films like the measured XPS data in the main text and fit them using equation 7. For these fits, which are also shown in Supplementary Fig. 1d, the fraction of SrO-termination and the atomic concentration of Sr, N_0^{Sr}, were allowed to vary like described in the main text. For each

case, we end up with the best fit with 100 % Sr-termination. The stoichiometry is summarized in Supplementary Table 1 along with the nominal stoichiometry for each film. For both highly-nonstoichiometric films with extended defects, we achieve a reasonable fit with a relative error of below 3.5 %. This indicates that extended defects can in fact lead to an overestimation or underestimation of the true cation ratio of a given film, as was indicated in the main text. At the same time, these worst case scenarios yield a comparably small relative error, especially when compared to the experimental uncertainty or the enormous effect pure termination effects can have (up to 17 %, compare 2 and main text). We therefore deem our analytical model as a very useful tool for the simultaneous determination of termination and stoichiometry.

References

1. Lee, J. H. *et al.* Dynamic layer rearrangement during growth of layered oxide films by molecular beam epitaxy. *Nat. Mater.* **13**, 879–883 (2014).
2. Zhang, L., Wett, D., Szargan, R. & Chassé, T. Determination of ZnO(0001) surface termination by x-ray photoelectron spectroscopy at photoemission angles of 0 and 70. *Surf. Interface Anal.* **36**, 1479–1483 (2004).

Supplementary Tables

Supplementary Table 1. Nominal film stoichiometry and best fit obtained with equation 7 from the main text.

Chemical Formula	True cation ratio $N_0^{Sr}/(N_0^{Sr}+N_0^{Ti})$	cation ratio $N_0^{Sr}/(N_0^{Sr}+N_0^{Ti})$ extracted from the fit
$SrTiO_3$	50.0 %	50.0 %
$Sr_{14}Ti_{13}O_{40}$	51.9 %	53.6 %
Sr_2TiO_4	66.7 %	68.6 %

Supplementary Figures

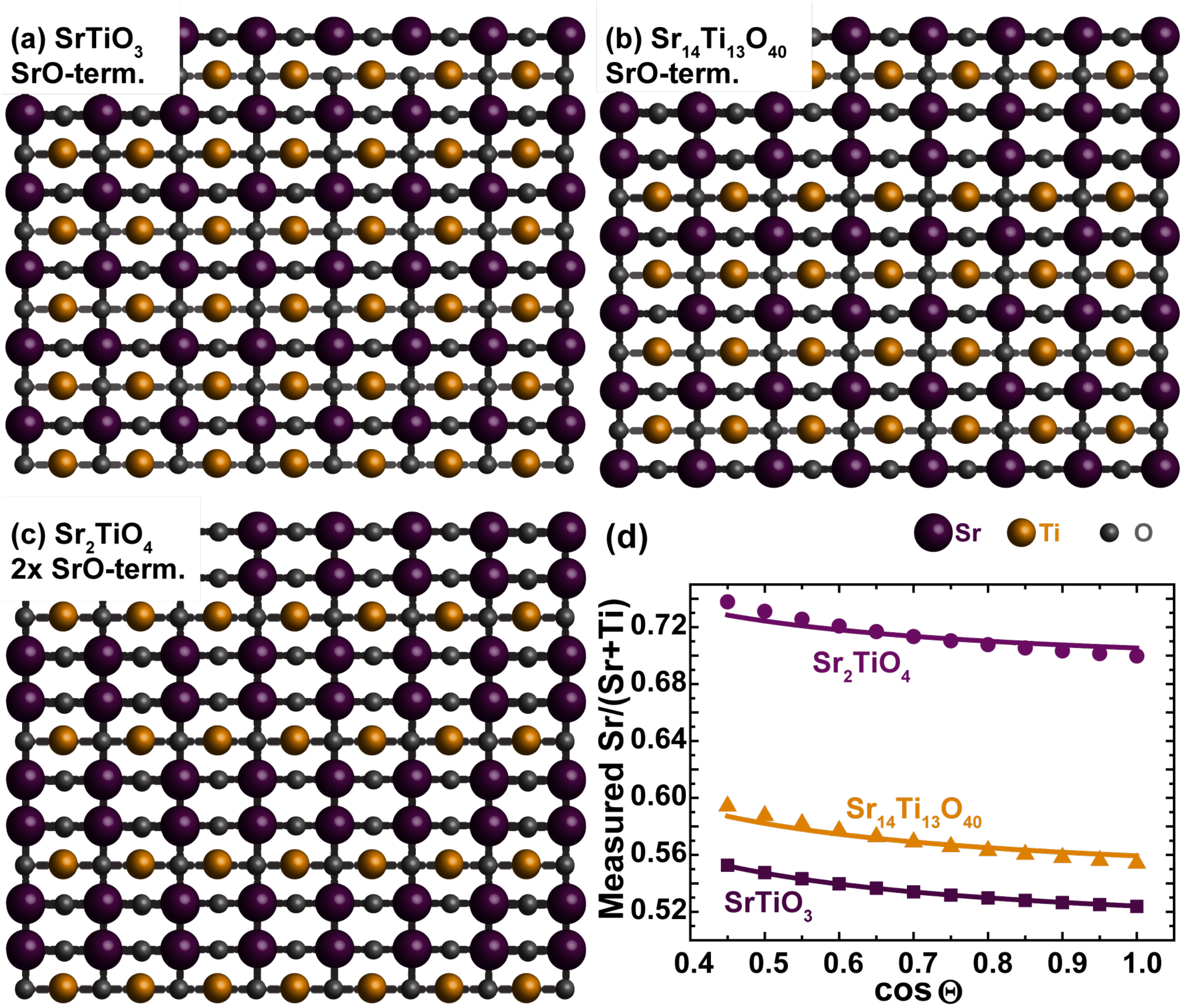

Supplementary Figure 1. Termination and stoichiometry determination for $SrTiO_3$ thin films with severe Sr excess. (a) Schematic illustration of a $SrTiO_3$ single crystals with SrO termination. (b) Schematic illustration of a $Sr_{14}Ti_{13}O_{40}$ single crystals with SrO termination and a double SrO layer burried one unit cell underneath the surface. (c) Schematic illustration of a Sr_2TiO_4 single crystals with a two-layer-SrO termination. (d) calculated cation intensity ratio (data points, compare Supplementary Note 1) as a function of the photoemission angle and fits (solid lines) according to equation 7 of the main text.
